# Dr. David W. Yandell

**Published:** 1898-06

**Authors:** 


### OBITUARY.

Dr. David W. Yandell, of Louisville, Kentucky, died at his residence in that city May 2, 1898, aged 71 years. Dr. Yandell was the son of the late Lunsford P. Yandell, and both father and son have been prominent professional figures in Kentucky since the early part of the century. Dr. D. W. Yandell was medical director on the staff of General Albert Sidney Johnston and ministered to the latter's mortal wound at Shiloh, April 6, 1862. He subsequently served with Generals Hardee and Kirby Smith until the close of the war. He then returned to Louisville, resumed the practice of his profession and became a teacher of surgery in the university of Louisville. He was one of the editors of the *American Practitioner and News*, though of late years, on account of his failing health, the editorial conduct of the journal has been entirely in the hands of his associate, Dr. H. A. Cottell. Dr. Yandell was a striking figure, a fine sportsman, a warm friend and a good hater. He will be missed in Louisville in professional and social circles. He is survived by a widow and two daughters residing at Louisville and one brother, who is a physician at El Paso, Texas.

